# CONNECTIONS: INTERGENERATIONAL DIGITAL STORYTELLING FOR CULTIVATING RESILIENCE ACROSS LIFESPAN

**DOI:** 10.1093/geroni/igae098.2853

**Published:** 2024-12-31

**Authors:** Vera Ansie, Crispin Mbamba

**Affiliations:** Kwame Nkrumah University of Science and Technology, Kumasi, Ashanti, Ghana; University at Albany, SUNY, Albany, New York, United States

## Abstract

This innovative study used intergenerational digital storytelling to explore resilience and fortitude across cultures. It aimed to develop and evaluate a culturally grounded intervention for synergistically cultivating resilience among older adults and youth through shared narratives and strengthened intergenerational ties. The participatory methods engaged older adult-youth dyads in one U.S. state (n=40) and Ghana (n=38) in a structured process of co-creating multimedia digital stories chronicling personal journeys of perseverance and overcoming hardship, with guided activities fostering reflection, reciprocal empathy, and intergenerational dialogue around resilience themes. Qualitative data from participant narratives, interviews, and observations were analyzed using constructivist grounded theory. Key findings highlighted storytelling’s transformative capacity for transmitting vital coping strategies, life lessons, and cultural wisdom between generations. For U.S. participants, salient themes included reframing challenges through optimism and self-reliance, while Ghanaian narratives emphasized fortitude rooted in spirituality, collectivism, and reverence for older adults’ resilience. Cross-cultural comparisons illuminated how social-ecological context distinctively shapes resilience processes. This study offers a novel, culturally responsive framework for designing intergenerational interventions that synergize resilience by providing hope through shared stories, modeling perseverance across ages, and honoring the fortitude of the human spirit, with implications for adaptation across diverse contexts, resilience theory, lifespan approaches, and culturally-grounded practice promoting well-being across generations.

